# Parental death in childhood and pathways to increased mortality across the life course in Stockholm, Sweden: A cohort study

**DOI:** 10.1371/journal.pmed.1003549

**Published:** 2021-03-11

**Authors:** Ayako Hiyoshi, Lisa Berg, Alessandra Grotta, Ylva Almquist, Mikael Rostila

**Affiliations:** 1 Clinical Epidemiology and Biostatistics, School of Medical Sciences, Örebro University, Örebro, Sweden; 2 Department of Public Health Sciences, Stockholm University, Stockholm, Sweden; 3 Department of Epidemiology and Public Health, University College London, London, United Kingdom; 4 Public Health, Department of Social Medicine, Osaka University Graduate School of Medicine, Osaka, Japan; 5 Centre for Health Equity Studies, Stockholm University/Karolinska Institutet, Stockholm, Sweden; London School of Hygiene and Tropical Medicine, UNITED KINGDOM

## Abstract

**Background:**

Previous studies have shown that the experience of parental death during childhood is associated with increased mortality risk. However, few studies have examined potential pathways that may explain these findings. The aim of this study is to examine whether familial and behavioural factors during adolescence and socioeconomic disadvantages in early adulthood mediate the association between loss of a parent at age 0 to 12 and all-cause mortality by the age of 63.

**Methods and findings:**

A cohort study was conducted using data from the Stockholm Birth Cohort Multigenerational Study for 12,615 children born in 1953, with information covering 1953 to 2016. Familial and behavioural factors at age 13 to 19 included psychiatric and alcohol problems in the surviving parent, receipt of social assistance, and delinquent behaviour in the offspring. Socioeconomic disadvantage in early adulthood included educational attainment, occupational social class, and income at age 27 to 37. We used Cox proportional hazard regression models, combined with a multimediator analysis, to separate direct and indirect effects of parental death on all-cause mortality.

Among the 12,582 offspring in the study (men 51%; women 49%), about 3% experienced the death of a parent in childhood. During follow-up from the age of 38 to 63, there were 935 deaths among offspring. Parental death was associated with an elevated risk of mortality after adjusting for demographic and household socioeconomic characteristics at birth (hazard ratio [HR]: 1.52 [95% confidence interval: 1.10 to 2.08, *p*-value = 0.010]). Delinquent behaviour in adolescence and income during early adulthood were the most influential mediators, and the indirect associations through these variables were HR 1.03 (1.00 to 1.06, 0.029) and HR 1.04 (1.01 to 1.07, 0.029), respectively. After accounting for these indirect paths, the direct path was attenuated to HR 1.35 (0.98 to 1.85, 0.066). The limitations of the study include that the associations may be partly due to genetic, social, and behavioural residual confounding, that statistical power was low in some of the subgroup analyses, and that there might be other relevant paths that were not investigated in the present study.

**Conclusions:**

Our findings from this cohort study suggest that childhood parental death is associated with increased mortality and that the association was mediated through a chain of disadvantages over the life course including delinquency in adolescence and lower income during early adulthood. Professionals working with bereaved children should take the higher mortality risk in bereaved offspring into account and consider its lifelong consequences. When planning and providing support to bereaved children, it may be particularly important to be aware of their increased susceptibility to delinquency and socioeconomic vulnerability that eventually lead to higher mortality.

## Abstract 2

In this cohort study, Ayako Hiyoshi and colleagues show associations between parental death in a child’s life and mortality risk later in that child’s life.

## Author summary

**Why was this study done?:**

- Experience of parental death during childhood has been associated with increased mortality in bereaved children.
- Bereavement in childhood has been associated with subsequent familial and behavioural difficulties and socioeconomic disadvantages over the life course.
- No study has examined which of these factors contribute to shaping a trajectory linking parental death to mortality in bereaved children.

**What did the researchers do and find?:**

- We followed a cohort of children born in Stockholm, Sweden, from birth to the age of 63.
- We conducted statistical analyses to assess the extent to which the association between childhood parental death (between age 0 and 12) and increased mortality in bereaved children by age 63 operated through familial and behavioural difficulties and socioeconomic disadvantages during adolescence and early adulthood.
- We found that childhood parental death was associated with an approximately 50% increased mortality risk in bereaved children.
- Delinquent behaviour in adolescence and low income during early adulthood appeared to mediate the association.

**What do these findings mean?:**

- When planning and providing support to bereaved children, it is particularly important to be aware of their increased susceptibility to delinquency and socioeconomic vulnerability that eventually lead to higher mortality.

## Introduction

Although the death of a parent in childhood is highly unexpected in Western societies, approximately 3% to 5% of children experience this loss [1–3]. As one of the most difficult life events for a child or adolescent may face [4], it can have long-term consequences over a lifetime and across generations. A body of research has shown that childhood parental death is associated with various health outcomes, including depression [5–7], self-inflicted injuries [8,9], suicide [10], asthma [11], higher body mass index [12], lower immune function [13], and an approximately 50% increased all-cause mortality risk [14–16]. Such adverse health effects may even carry over to the next generation; higher likelihood of premature birth and lower birth weight have been observed in bereaved children’s male offspring [17].

Despite the accumulation of evidence for negative health consequences, few studies have examined potential pathways linking parental death to mortality or quantified the extent of specific mediating paths. Childhood parental death is a traumatic and stressful life events and has been considered to disrupt the developmental trajectories and impair child’s ability to cope with adversity [18], potentially resulting in behavioural and emotional problems, such as misbehaviour and anger [19]. Indeed, parental death has been found to be associated with an increased rate of behavioural problems [20], delinquent behaviour [21], and externalizing problems [22], including substance abuse [23], violent crime [24], and risky sexual behaviour [20]. An increased risk for mental health problems in the surviving parent can relate to the quality of care that children receive following the death and thus have also implications for the child rearing environment [18,25]. Furthermore, the death of a parent can result in financial decline of the family [26], and socioeconomic disadvantage in childhood is associated with various negative health consequence across the life course [18]. Both delinquency and socioeconomic disadvantage have been shown to be linked to low educational aspiration and school performance [27,28], which in turn are likely to lead to socioeconomic disadvantages in adulthood, such as unstable employment and lower income [28–30]. A number of studies have shown that adult socioeconomic disadvantage is associated with higher mortality risk [31]. Delinquency may also relate to higher mortality risk due to greater exposure to stressful or hazardous experiences [32] and unhealthy behaviour [33]. However, the extent to which such chain of adversities following the parental death mediate the association with increased mortality risk in the bereaved child has not been examined.

Therefore, the aim of this study is to examine the extent to which both behavioural and familial characteristics and adult socioeconomic factors across life course may mediate the association between parental death at age 0 to 12 and all-cause mortality by the age of 63. Given that previous studies have provided inconclusive evidence regarding differences by the sex of the deceased parent and/or the child [34], we will also examine sex differences in the consequences of childhood parental death and patterns of mediating paths.

## Methods

### Data and sample

Using the Stockholm Birth Cohort Multigenerational Study, which includes 14,608 children who were born in 1953 and lived in the greater Stockholm metropolitan area at age 10 [35], a cohort study was conducted. Participants were followed up through surveys and national registers until 2016. Details of how the cohort was set up and followed can be found elsewhere [35]. Information from childhood and adolescence is available by the following 3 periods: period 1 (age 0 to 6, 1953 to 1959), period 2 (age 7 to 12, 1960 to 1965), and period 3 (age 13 to 19, 1966 to 1972). We focused on parental death occurring during periods 1 to 2, while potential mediating variables were obtained from period 3 and early adulthood (age 27 to 37 years old, 1980 to 1990). Information on all-cause mortality refers to the remaining period (age 38 to 63, 1991 to 2016).

Among 14,608 participants, those who died (*n* = 253) or emigrated (*n* = 738) before the start of the follow-up of the present study (1991) were excluded. Furthermore, participants who experienced parental death in period 3 (*n* = 519), lost both parents (*n* = 5), or had missing data in relevant variables (*n* = 511)—mostly in childhood and early adulthood socioeconomic variables—were excluded, leaving 12,582 participants eligible for analysis. The data were anonymised before use. The regional ethical review board of Lund approved the study (2019–06175).

### Variables

#### Exposure: Parental death at age 0 to 12 (1953 to 1965)

Information about parental death was derived from 2 sources: Swedish national registers and local Social Registers. Using the Multi-Generation Register, we identified parent–offspring dyads and, by linking it to the Cause of Death Register, the date of death of a parent was ascertained. The register includes virtually all deaths in Sweden, with cause of death being recorded for 96% of all deaths [36]. Parents were identified through the Multi-Generation Register for more than 95% of children born in 1953 in Sweden [37]. The Social Registers were used to supplement the identification of parental death. They were held by each municipality in the greater Stockholm metropolitan area and contained information on families and participants who had received any services from local social services [38]. Parental deaths known by the Child Welfare Committee were recorded in the register, and the information was extracted in 1972. Ninety-one percent of parental deaths overlapped in the 2 registers.

#### Outcome: All-cause mortality at age 38 to 63 (1991 to 2016)

The date of death of a participant was identified using the Cause of Death Register. In order to examine associations for concordant or discordant causes of death between the deceased parent and the offspring, causes of death were further separated into unnatural and natural causes. Unnatural causes included suicide and other external causes, such as accidents, and were identified using the Swedish edition of International Classification of Diseases (ICD) 7 E800-E999, ICD8 E807-E999, ICD9 E800-E999, ICD10 V01-Y98 [39,40]. Natural causes included all other causes of death.

### Covariates

The sex of the offspring was specified as male or female. It has been found that birth order can associate with behaviour [41] and psychological resilience [42]; therefore, a dichotomous variable was created to indicate if the child was first-born or later-born. Social class in 1953 was based on father’s occupation and classified into 5 groups according to the then classification system: upper middle class and higher; lower middle class including officials and nonagricultural workers; lower middle class including self-employed; working class, skilled workers; and working class, unskilled workers.

### Mediators

#### Familial and behavioural mediators, age 13 to 19 (1966 to 1972)

Information on potential mediators during adolescence was extracted from the Social Registers in 1972. Parents’ psychiatric problems included the receipt of psychiatric treatment or being depressed. Incidence of drunkenness and alcohol abuse in the surviving parent was recorded in the register if they were found guilty of drunkenness as a misdemeanour or drunk driving, or identified as an alcoholic. Receipt of social assistance was recorded in Swedish krona. Delinquency was measured based on the number of welfare support decisions made by the Child Welfare Committee to provide assistance, supervision, or placement into social care due to, for example, stealing, violence, substance abuse, and other offences, with the value being right-truncated at the maximum of 9. After assessing the model fit, apart from delinquency, the variables were dichotomised. Delinquency showed departure from linearity and was modelled using linear spline with knots at scores 2 and 5.

#### Socioeconomic mediators, age 27 to 37 (1980 to 1990)

Information on educational attainment was obtained from the Longitudinal Integration Database for Health Insurance and Labour Market Studies in 1990, while information on social class and income was obtained from the Population and Housing Censuses in 1980 and 1985, respectively. Educational attainment was based on the highest attained level and classified into 3 groups: less than secondary (approximately <10 years of education), secondary (10 to 12 years), and postsecondary education (≥13 years). Social class was based on occupation and categorised into 7 groups [43]. Individual disposable income was reported in Swedish krona (per 1,000 krona). Income was included in the analyses as a continuous variable, while educational attainment and social class were treated as categorical variables.

### Statistical analysis

The characteristics of participants were assessed by percentages, means, and standard deviations. The distribution of data between offspring with and without a parental death was compared using chi-squared test for categorical variables and Wilcoxon rank-sum test for continuous variables because of their skewed distribution. Cox proportional hazard regression and a multimediator mediation analysis were used to assess association and separate direct and indirect effects [44]. The follow-up started in 1991 (age 38)—after the assessment of adulthood mediators—and ended at the time of death, emigration, or 31 December 2016, whichever occurred first. We computed hazard ratios (HRs) and 95% confidence intervals (CIs). The focus on the assessment of mediating paths was preplanned, and an extract from relevant documents can be found in S1 Protocol. The proportional hazard assumption was assessed both graphically and using Schoenfeld residuals and was verified for all variables, except income.

We performed a series of Cox models including/excluding mediating variables. Likelihood ratio tests were used to assess which of the mediators were the most influential. Model 1 is unadjusted, Model 2 is adjusted for confounding factors, Models 3 to 7 included familial and behavioural mediators, and Models 8 to 11 included further socioeconomic mediators. The role of offspring’s sex as a potential moderator of the association between parental death and mortality was investigated by testing an interaction term in the unadjusted model. We also investigated differences in the association by the sex of deceased parent by reclassifying the variable for parental death into 3 groups—no parental death, paternal death, and maternal death—and including it in the unadjusted model. Furthermore, estimates stratified by the sex of the deceased parent and of the offspring as well as by a combination of these 2 variables were obtained for comparability with preceding literature.

**Fig 1:**
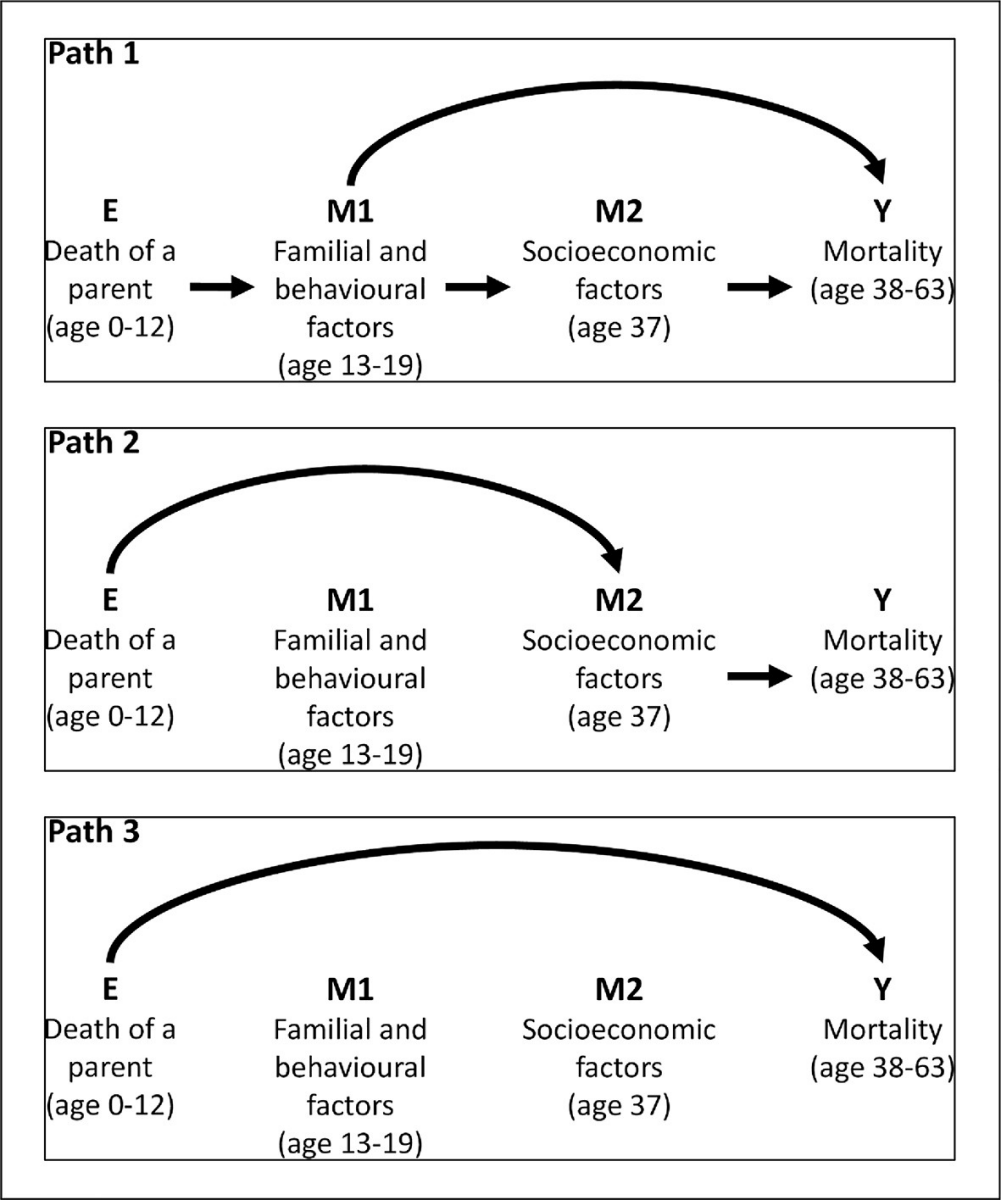
Potential mediating (paths 1–2) and direct (path 3) paths linking childhood loss of a parent and mortality.

To obtain mediated effect sizes, a multimediator mediation analysis was conducted by including the most influential mediators [44]. Path 1 is the effect of E (parental death) that involves M1 (a familial/behavioural mediator) and possibly also M2 (a socioeconomic mediator) (Fig 1), and Path 2 is the effect of E that involves only M2. Path 3 is the direct path that did not involve any of the mediators. While the direct effect is interpreted as the effect of the exposure on the outcome that is not explained by exposure-induced changes in the mediators, an indirect effect through a specific mediator (or set of mediators) is interpreted as the effect of the exposure on the outcome which can be explained by exposure-induced changes of the mediator(s) [45,46]. These analyses were carried out according to a preplanned protocol (S1 Protocol).

Three analyses suggested by the peer reviewers have been conducted. First, a generalized structural equation model (GSEM) was fitted as an alternative approach to investigate associations between variables and mediating paths. For this analysis, logistic regression was used to model parental death, linear regressions were used to model delinquency and income, multinomial logistic regressions were used to model education and social class (highest educational group and social class were chosen as reference categories), and parametric survival regression was used to model mortality. We used Weibull hazard distribution for survival regression, as it showed the best fit compared with other distributional forms according to Akaike and Bayesian Information Criteria. Estimates for multinomial and survival regressions were exponentiated and displayed as relative risks and HR, respectively. Second, because living environment and psychiatric or genetic characteristics associated with relatively early parental death may also predispose offspring to a higher risk of mortality, HRs for concordant or discordant causes of death between the deceased parent and offspring were estimated [6,14,40,47,48]. Unnatural and natural causes of death in offspring were analysed separately, and HRs were estimated using a variable separating a parental death into unnatural and natural causes, with no parental death as the reference category. In the analysis of unnatural causes of death in offspring, natural causes were censored, and vice versa. A Wald test was conducted to test the equality of HRs for unnatural and natural causes of parental death. Offspring whose parent had died from unidentified causes were excluded from this analysis (*n* = 18). Third, a Cox model with follow-up starting from 1973 (age 20) was fitted to include deaths occurred during early adulthood (age 20 to 37). In this analysis, only the mediator M1, i.e., delinquency, was involved because data on socioeconomic conditions were not consistently available for this age range.

All analyses were conducted using Stata/SE version 15 and R version 3.6.1. *p*-Values lower than 0.05 were considered statistically significant. This study is reported as per the Strengthening the Reporting of Observational Studies in Epidemiology (STROBE) guideline (S1 Checklist).

## Results

During a median follow-up of 26.0 years, there were 935 deaths among 12,582 participants (men 51%; women 49%). Approximately 3% had experienced parental death at age 0 to 12 (Table 1). Those who experienced parental death were more likely to be later-born than first-born. The distribution of parental social class tended to be similar between families with and without a parental death. There were small differences in the distribution of psychiatric or alcohol problems between families with and without a parental death, but households in which the father died were more likely to receive social assistance. Experiences after parental death appeared to differ by the sex of the deceased parent or the offspring (T1 in S1 Table). Delinquency was generally more frequent in male offspring and among adolescents who experienced a parental death. In adulthood, male offspring who had lost a parent tended to have lower educational attainment and were more likely to be unskilled workers and to have slightly lower income than those who had not experienced a parental death. This pattern was not consistently observed among female offspring.

**Table 1:** Characteristics of offspring by the death of a parent (age 0–12).

	No parental death	Parental death			*p*-value
		Death of a parent	Death of the father	Death of the mother	
	*N* (%)^a^	*N* (%)^a^	*N* (%)^a^	*N* (%)^a^	
	12,708 (97.1)	385 (2.9)	270 (2.1)	115 (0.9)	
**Sex of offspring**					
Male	6,511 (51.2)	198 (51.4)	142 (52.6)	56 (48.7)	
Female	6,197 (48.8)	187 (48.6)	128 (47.4)	59 (51.3)	0.940
**Birth order**					
First-born	7,301 (57.5)	172 (44.7)	128 (47.4)	44 (38.3)	
Later-born	5,407 (42.6)	213 (55.3)	142 (52.6)	71 (61.7)	<0.001
**Parental social class**					
Upper middle class and higher	1,647 (13.0)	61 (15.8)	43 (15.9)	18 (15.7)	
Lower middle class, including officials and nonagricultural workers	3,953 (31.1)	118 (30.7)	84 (31.1)	34 (29.6)	
Lower middle class, including self-employed	741 (5.8)	27 (7.0)	18 (6.7)	9 (7.8)	
Working class, skilled workers	3,579 (28.2)	84 (21.8)	58 (21.5)	26 (22.6)	
Working class, unskilled workers	2,396 (18.9)	84 (21.8)	58 (21.5)	26 (22.6)	0.034
Missing	392 (3.1)	11 (2.9)	9 (3.3)	2 (1.7)	
**Mediators**					
**Familial and behavioural factors (age 13–19)**					
** Psychiatric problems in parents/surviving parent**					
No	12,358 (97.3)	374 (97.1)	262 (97.0)	112 (97.4)	
Yes	350 (2.8)	11 (2.9)	8 (3.0)	3 (2.6)	0.903
** Alcohol problems in parents/surviving parent**					
No	12,393 (97.5)	382 (99.2)	269 (99.6)	113 (98.3)	
Yes	315 (2.5)	3 (0.8)	1 (0.4)	2 (1.7)	0.033
** Receipt of social assistance**					
No	11,376 (89.5)	322 (83.6)	219 (81.1)	103 (89.6)	
Yes	1,332 (10.5)	63 (16.4)	51 (18.9)	12 (10.4)	<0.001
**Delinquency**^b^					
Mean (SD), 5th to 95th percentiles	0.2 (0.7), 0 to 1	0.3 (0.78), 0 to 2	0.3 (0.7), 0 to 2	0.4 (0.8), 0 to 2	0.021
**Socioeconomic factors (age 27–37)**					
** Educational attainment**					
Postsecondary	4,668 (36.7)	137 (35.6)	95 (35.2)	42 (36.5)	
Upper secondary	5,546 (43.6)	167 (43.4)	118 (43.7)	49 (42.6)	
Less than secondary	2,412 (19.0)	79 (20.5)	55 (20.4)	24 (20.9)	0.739
Missing	82 (0.7)	2 (0.5)	2 (0.7)	0	
** Social class**					
Professional	1,115 (8.8)	28 (7.3)	23 (8.5)	5 (4.4)	
Business owner	420 (3.3)	12 (3.1)	9 (3.3)	3 (2.6)	
Midlevel office worker	2,377 (18.7)	67 (17.4)	45 (16.7)	22 (19.1)	
Lower-level office worker	2,326 (18.3)	48 (12.5)	35 (13.0)	13 (11.3)	
Skilled production/service worker	1,621 (12.8)	50 (13.0)	34 (12.6)	16 (13.9)	
Unskilled production/service worker	2,223 (17.5)	87 (22.6)	61 (22.6)	26 (22.6)	
Other^c^	2,600 (20.5)	93 (24.2)	63 (23.3)	30 (26.1)	0.012
Missing	26 (0.2)	0	0	0	
** Income**					
Mean (SD), 5th to 95th percentiles (per 1,000 Swedish krona)	95.3 (53.0), 5.4 to 184.8	89.3 (55.3), 0.2 to 162.9	91.5 (57.8), 0.3 to 164.3	84.0 (48.7), 0 to 159.0	0.034
Missing	38 (0.3)	0	0	0	

^a^Delinquency and income in adulthood are continuous variables and mean (SD) and 5th and 95th percentiles are displayed.

^b^Delinquency was defined by the number of welfare support decisions by the Child Welfare Committee from age 13 to 19. Values ranged from 0 to 9.

^c^Other included unclassified employees, pensioners, homemakers (male and female), students, part-time workers (less than 16 h/week), and other unspecified groups.

*p*-value refers to chi-squared test for categorical variables and Wilcoxon rank-sum test for continuous variables. All tests were conducted after excluding missing data.

Parental death was associated with a 50% elevated risk of mortality in the unadjusted Cox model (Model 1) (Table 2 and T2 in S1 Table). This risk did not decrease after adjustment for covariates (Model 2) or mediating variables reflecting parental psychiatric or alcohol problems, or receipt of social assistance (Model 6). However, the inclusion of delinquency led to a large attenuation of the association (Model 7), while the removal of other mediating variables during adolescence, including parental psychiatric and alcohol problems and receipt of social assistance from Model 7 did not statistically significantly change the model fit (likelihood ratio test *p*-value = 0.868). Regarding the socioeconomic mediators, all variables significantly improved the model (likelihood ratio tests *p*-values < 0.05), and income followed by occupation attenuated the association the most (Models 8 to 11).

**Table 2:** Hazard ratios for the association between the death of a parent during childhood (age 0–12) and all-cause mortality (age 38–63).

	Event^a^/*N*	Model 1	Model 2	Model 6	Model 7	Model 11
		HR (95% CI), *p*-value	HR (95% CI), *p*-value	HR (95% CI), *p*-value	HR (95% CI), *p*-value	HR (95% CI), *p*-value
**The death of a parent**	935/12,582					
No parental death	895/12,210	Reference	Reference	Reference	Reference	Reference
Parental death	40/372	1.50 (1.09, 2.05), 0.013	1.52 (1.10, 2.08), 0.010	1.49 (1.09, 2.05), 0.013	1.38 (1.01, 1.90), 0.046	1.32 (0.96, 1.81), 0.091

^a^Event: Death of offspring.

The numbering of models corresponds to T2 in S1 Table.

Model 1: Unadjusted.

Model 2: Adjusted for covariates (sex of offspring, birth order, parental social class).

Model 6: Model 2 + psychiatric problems and alcohol problems in parents/surviving parent + receipt of social assistance (age 13–19).

Model 7: Model 2 + psychiatric problems and alcohol problems in parents/ surviving parent + receipt of social assistance + delinquency (age 13–19).

Model 11: Model 7 + education (age 37) + social class (age 27) + income (age 32).

The estimated interaction parameter showed that the association was weaker in females than in males (T3 in S1 Table). However, the difference was not statistically significant (*p*-value = 0.435). Furthermore, compared to paternal death, maternal death was less strongly associated with offspring’s mortality, although this difference was not statistically significant (*p*-value = 0.705) (T4 in S1 Table). In accordance with the above analyses, when the analyses were stratified by the sex of offspring, HRs were higher in men whereas no significant association was shown in women, and paternal death was associated with higher mortality than maternal death (T5 in S1 Table). As in the nonstratified analyses, the association was most attenuated by delinquency and income. When we further stratified the analysis by parental and offspring sex jointly, male offspring who had lost a mother showed a more than 2-fold risk of mortality compared with male offspring who had not lost a parent. However, the number of exposed offspring and outcome event was heavily reduced in each stratum.

The mediation analysis focusing on delinquency and income in adulthood showed that, of the total effect of HR 1.44 (95% CI 1.05, 1.98), the death of a parent increased the risk of mortality through delinquency (Path 1) by a small effect size but was statistically significantly (HR 1.03 [95% CI 1.00, 1.06]) (Table 3). A path through lower income in adulthood also made a small but statistically significant impact (HR 1.04 95% CI [1.01, 1.07]) (Path 2). After accounting for these paths, the direct association between parental death and mortality was reduced and became nonsignificant (HR 1.35 [95% CI 0.98, 1.85]) (Path 3). When the same analysis was performed for the death of fathers and mothers separately, indirect paths 1 and 2 were not statistically significant, but these paths were close to 5% significance in relation to mother’s death, although the total effect was not significant (T6 in S1 Table). When the analysis was stratified by sex of offspring, we found that among males, the path through adulthood income (path 2) was significant, and the path through delinquent behaviour and possibly adult income (path 1) was marginally significant. No significant direct or indirect paths were detected among females.

**Table 3:** Multimediator analysis for Cox proportional hazard regression for the association between the death of a parent in childhood (age 0–12) and all-cause mortality (age 38–63).

	Death of a parent
	HR (95% CI), *p*-value
**Total effect of exposure**	1.44 (1.05, 1.98), 0.026
**Path 1**: The effect of E that involves M1 (delinquency) and possibly M2 (adulthood income)	1.03 (1.00, 1.06), 0.029
**Path 2**: The effect of E on Y only mediated through adulthood income	1.04 (1.01, 1.07), 0.029
**Path 3**: The effect of E on Y not via pathways involving delinquency or income	1.35 (0.98, 1.85), 0.066

In the mediation analysis, it was not possible to model the nonlinear relationship between delinquency and mortality, thus the association was treated as linear.

In the GSEM analysis, parental psychiatric and alcohol problems and the receipt of social assistance did not significantly improve the model (likelihood ratio test *p*-value = 0.987); therefore, they were not included. All other variables showed association with at least one variable in either the direct or indirect paths between parental death and mortality in offspring. Statistically significant coefficients relevant to these paths and interpretations are shown in S1 Fig, and all coefficients are shown in T7 in S1 Table. As in the previously conducted mediation analysis, there was no direct path from parental death to mortality and the association was linked through indirect paths involving delinquency and income. Unlike the mediation analysis, however, there was no effect of parental death that was linked only through income (parental death → income → mortality). This difference may be because the mediation analysis did not include education and occupation in the model.

In the examination of concordant and discordant causes of death between parents and offspring, CIs tended to be wide due to low statistical power (Table 4). Although the highest HR was observed when both parents and offspring died from a concordant unnatural cause (HR 2.01 [95% CI 0.50, 8.17]), the HR for discordant causes (unnatural causes in parent and natural causes in offspring) was similar in magnitude (HR 1.78 [95% CI 0.98, 3.23]). The HRs for natural or unnatural causes of parental death was not statistically significantly different in unnatural causes of death in offspring (Wald test *p*-value = 0.560). Similarly, the HRs did not significantly differ when the outcome was offspring’s natural death (Wald test *p*-value = 0.470). Therefore, there was no clear evidence that HRs for concordant and discordant causes of death were significantly different.

**Table 4:** Distribution and hazard ratios for the association between the cause of death of a parent during childhood (age 0–12) and cause of death in offspring (age 38–63).

UNNATURAL CAUSE OF DEATH IN OFFSPRING		
	Events/*N*	HR (95% CI), *p*-value
Not parental death	127/12,210	Reference
Parental death due to unnatural causes (a)	2/101	2.01 (0.50, 8.17), 0.327
Parental death due to natural causes (b)	3/253	1.18 (0.38, 3.72), 0.775
*p*-value for Wald test, a = b		0.560
**NATURAL CAUSE OF DEATH IN OFFSPRING**		
Not parental death	768/12,210	Reference
Parental death due to unnatural causes (a)	11/101	1.78 (0.98, 3.23), 0.058
Parental death due to natural causes (b)	21/253	1.37 (0.89, 2.11), 0.159
*p*-value for Wald test, a = b		0.476

The analysis was conducted for 12,564 offspring after excluding 18 offspring for whom cause of parental death was unknown. HRs were adjusted for sex of offspring, birth order, and parental social class in 1953.

The analysis in which the follow-up started in 1973 (age 20) found a few deaths in the bereaved offspring occurring in their 20s and early 30s (T8 in S1 Table). Parental death showed a statistically significant 38% increased risk of mortality after adjustment for covariates (Model 2 in T8 in S1 Table). The association was slightly weaker than the one observed when the follow-up started in 1991 (age 38), although there was no evidence of the violation of proportional hazard assumption (Schoenfeld residual test *p*-value = 0.260). Parental psychiatric and alcohol problems and the receipt of social assistance did not change the HR noticeably (Model 6). When delinquency was included, the HR showed obvious attenuation and parental death became no longer associated with mortality (Model 7). This may be because delinquency may have had a stronger association with deaths occurring during early adulthood.

## Discussion

Using data gathered over 6 decades, we examined the paths linking the experience of the death of a parent at the age of 12 or under to all-cause mortality between the age of 38 and 63 years. Parental death was associated with an approximately 50% higher risk of mortality in offspring. The association was mediated through a chain of disadvantages including delinquency in adolescence and lower income in early adulthood, which was particularly evident in men.

While a large number of people manage, recover, and even thrive after traumatic events [49], early adverse experiences can place individuals on disadvantaged life trajectories that are associated with increased mortality [50,51]. From a life-course perspective, particularly for males, our findings support the chain of risk model, that exposure is associated with the risk of subsequent negative events, and each of these factors are in turn associated with increased mortality risk [52]. The death of a parent during childhood is a traumatic and stressful life event and has been found to increase susceptibility to delinquent behaviour [21,24]. Those engaging in delinquent behaviour face a higher rate of negative life stressors and disrupted lives [33,53]. Progression to higher education and obtaining advantageous jobs are less common in youth with experience of family disruption, including the death of a parent [27,54], and socioeconomic disadvantage has been linked to altered stress response [55], immune and metabolic physiology and an increased predisposition to inflammation [56], which relates to the risk of major causes of death [57]. Impaired functioning of the surviving parent has been associated with the child’s development [5,25,26]. However, we observed little impact of alcohol abuse and psychiatric problems in the surviving parent on the association with mortality in offspring.

Previous studies have been inconclusive regarding differences by the sex of the deceased parent and/or the offspring [34]. Although the difference was not statistically evident, we found a stronger association between paternal death and mortality in offspring than maternal death. This finding diverges from previous Swedish studies which have shown that maternal death during the period from childhood to early adolescence had a similar or even higher magnitude association than paternal death in cohorts which grew up in the 1970s and 1980s. These studies investigated the risk of mortality in offspring in the period immediately following parental death [15] and depression treatment in young adulthood [6]. In the years 1953 to 1965 when our study participants grew up, the male-breadwinner model prevailed, so the death of the father could possibly have had a stronger impact on the child’s economic and social circumstances, educational attainment, and socioeconomic trajectories in their life course. Since then, female participation in the labour market has risen dramatically, from 49% in 1963 to 84% in 1982 [58]. Family structures and responsibilities have changed, leading to a more equal sex distribution in terms of material and economic resources and thus the overall life chances to which parents contribute. This might have increased the relative importance of the mother’s death on mortality in bereaved offspring in more recent cohorts. With respect to sex of offspring, we did not observe an association in women, although there was no statistical evidence that HRs differ by the sex of offspring. Lack of statistical significance for both the interaction test and the association in women may be partly because the cohort was still relatively young and the number of deaths in women was half of that in men, leading to low statistical power.

The strengths of our study include the use of material from a unique birth cohort, encompassing decades of data and including a wide range of familial, behavioural, and socioeconomic variables across the life course for a fairly large number of participants representative of children born in Stockholm, Sweden in 1953.

Some limitations of this study need to be noted. First, because the exposure was a relatively rare event, the statistical power was low and coefficients and standard errors could have been biased in some of the stratified analyses, particularly in the analyses stratified by sex of both parent and child. Therefore, it is recommended that the analysis by sex of parents and offspring are replicated by a study with a larger sample size. Second, there is a possibility that the observed associations may be due to unmeasured confounding. The death of parent and offspring may have common prior causes, such as genetic, social, and behavioural factors. As in the previous literature [6,14,40,47,48], to assess this possibility, we examined deaths due to specific causes (natural and unnatural causes) concordant or discordant between parents and offspring. We found that concordant causes of death did not consistently show higher association than discordant causes of death. The highest HR was observed for concordant unnatural causes of death in parents and offspring, which may imply genetic and shared environmental confounding [59]. Nevertheless, the HR for discordant cause of death (unnatural cause of death in the deceased parent and natural cause of death in offspring) was not markedly lower. Therefore, we consider that the associations may be partly, but may not be entirely, explained by unmeasured confounding. Third, because socioeconomic factors were obtained from censuses in 1980, 1985, and 1990, we started follow-up in 1991 when the offspring were aged 38 years. The mortality rate of delinquent young people tends to be high even in young age [60]. In our data, delinquency in adolescence was recorded in 83 (41%) of 202 deaths that occurred between the age of 20 and 37. Conditioning on survival until 1991 may have introduced collider selection bias [61]. In the analysis in which follow-up started in 1973, the association between parental death and mortality became weaker when delinquency was adjusted for; this may indicate that delinquency may be particularly relevant to early death. Fourth, although the direct path (path 3) was statistically nonsignificant, given the small effect size of the mediating paths and the relatively high magnitude of the direct path, other paths that were not included in the present study may still be relevant, such as increased susceptibility to stress in offspring who have experienced parental loss [62]. Furthermore, the mediation analysis assumes that there is no confounder between the mediator and outcome relationship that is affected by the exposure. However, the variables in adolescence and the early adulthood were probably influenced by the exposure and mutually associated; therefore, the results of the mediation analysis rely on the assumption of the absence of such relationships. Finally, the generalizability of our findings could be limited. The experience of losing a parent and its health consequences should be interpreted in relation to the societal and historical context where our birth cohort grew up [63]. Differences in welfare systems and support functions for bereaved families in other societies or society today may result in stronger (or weaker) associations between parental death and mortality in offspring and roles of mediating variables.

### Conclusions

The present study showed that parental death is associated with increased mortality risk in offspring. Delinquency during adolescence and lower income in the early adulthood were involved in the pathways leading to increased mortality risk among individuals who had experienced parental death, especially men. Professionals working with bereaved children should take the higher mortality risk in bereaved offspring into account. When planning and providing support to bereaved children, it is particularly important to be aware of their increased susceptibility to delinquency and socioeconomic vulnerability that eventually lead to higher mortality.

## Supporting information

S1 Checklist STROBE Statement checklist of items for cohort studies. (DOCX)

S1 Protocol Extracts from the funding proposal and the protocol of analysis. (DOCX)

S1 Table T1 to T8 supplementary tables. (DOCX)

S1 Fig Coefficients, risk ratios, and hazard ratios estimated using GSEM linking parental death (age 0–12) to mortality (age 38–63). (DOCX)

## Abbreviations

CI: confidence interval
GSEM: generalized structural equation model
HR: hazard ratio
ICD: International Classification of Diseases
STROBE: Strengthening the Reporting of Observational Studies in Epidemiology
