# Supplementary material for: Parental death in childhood and pathways to increased mortality across the life course in Stockholm, Sweden: A cohort study
Source: PLoS Med. 2021 Mar 11;18(3):e1003549. doi: 10.1371/journal.pmed.1003549 (PMC7951838; doi:10.1371/journal.pmed.1003549)
Supplement: S1 Checklist — (DOCX) [file pmed.1003549.s001.docx]

**S1 Checklist STROBE Statement checklist of items for cohort studies.**

|  | Item No | Recommendation | Location |
| --- | --- | --- | --- |
| **Title and abstract** | 1 | (*a*) Indicate the study’s design with a commonly used term in the title or the abstract | Title, abstract |
|  |  | (*b*) Provide in the abstract an informative and balanced summary of what was done and what was found | Abstract, Author summary |
| Introduction | | | |
| Background/rationale | 2 | Explain the scientific background and rationale for the investigation being reported | Introduction |
| Objectives | 3 | State specific objectives, including any prespecified hypotheses | 3^rd^ paragraph in Introduction |
| Methods | | | |
| Study design | 4 | Present key elements of study design early in the paper | 1^st^ paragraph in Data and sample |
| Setting | 5 | Describe the setting, locations, and relevant dates, including periods of recruitment, exposure, follow-up, and data collection | 1^st^ paragraph in Data and sample |
| Participants | 6 | (*a*) Give the eligibility criteria, and the sources and methods of selection of participants. Describe methods of follow-up | 2^nd^ paragraph in Data and sample |
|  |  | (*b*) For matched studies, give matching criteria and number of exposed and unexposed | Not applicable |
| Variables | 7 | Clearly define all outcomes, exposures, predictors, potential confounders, and effect modifiers. Give diagnostic criteria, if applicable | Variables section in Methods |
| Data sources/ measurement | 8* | For each variable of interest, give sources of data and details of methods of assessment (measurement). Describe comparability of assessment methods if there is more than one group | Variables section in Methods |
| Bias | 9 | Describe any efforts to address potential sources of bias | 4^th^ to 6^th^ paragraphs in Statistical analysis |
| Study size | 10 | Explain how the study size was arrived at | 2^nd^ paragraph in Data and sample |
| Quantitative variables | 11 | Explain how quantitative variables were handled in the analyses. If applicable, describe which groupings were chosen and why | Variables section in Methods and Statistical analysis |
| Statistical methods | 12 | (*a*) Describe all statistical methods, including those used to control for confounding | Statistical analysis |
|  |  | (*b*) Describe any methods used to examine subgroups and interactions | 2^nd^ paragraph in Statistical analysis |
|  |  | (*c*) Explain how missing data were addressed | 2^nd^ paragraph in Data and sample |
|  |  | (*d*) If applicable, explain how loss to follow-up was addressed | 1^st^ paragraph in Statistical analysis |
|  |  | (*e*) Describe any sensitivity analyses | 4^th^ paragraph in Statistical analysis |
| Results | | |  |
| Participants | 13* | (a) Report numbers of individuals at each stage of study—eg numbers potentially eligible, examined for eligibility, confirmed eligible, included in the study, completing follow-up, and analysed | 2^nd^ paragraph in Data and Sample |
|  |  | (b) Give reasons for non-participation at each stage | 2^nd^ paragraph in Data and Sample |
|  |  | (c) Consider use of a flow diagram | Not used |
| Descriptive data | 14* | (a) Give characteristics of study participants (eg demographic, clinical, social) and information on exposures and potential confounders | Table 1, 1^st^ paragraph in Results |
|  |  | (b) Indicate number of participants with missing data for each variable of interest | Table 1 |
|  |  | (c) Summarise follow-up time (eg, average and total amount) | 1^st^ paragraph in Results |
| Outcome data | 15* | Report numbers of outcome events or summary measures over time | Table 2 |
| Main results | 16 | (*a*) Give unadjusted estimates and, if applicable, confounder-adjusted estimates and their precision (eg, 95% confidence interval). Make clear which confounders were adjusted for and why they were included | Tabl2, T2 & T5 in S1 Table |
|  |  | (*b*) Report category boundaries when continuous variables were categorized | Not applicable |
|  |  | (*c*) If relevant, consider translating estimates of relative risk into absolute risk for a meaningful time period | Not conducted |
| Other analyses | 17 | Report other analyses done—eg analyses of subgroups and interactions, and sensitivity analyses | 3^rd^ and 5^th^ to 7^th^ paragraphs in Results |
| Discussion | | | |
| Key results | 18 | Summarise key results with reference to study objectives | 1^st^ paragraph in Discussion |
| Limitations | 19 | Discuss limitations of the study, taking into account sources of potential bias or imprecision. Discuss both direction and magnitude of any potential bias | 5^th^ paragraph in Discussion |
| Interpretation | 20 | Give a cautious overall interpretation of results considering objectives, limitations, multiplicity of analyses, results from similar studies, and other relevant evidence | 2^nd^ paragraph in Discussion |
| Generalisability | 21 | Discuss the generalisability (external validity) of the study results | 5^th^ paragraph in Discussion |
| Other information | | | |
| Funding | 22 | Give the source of funding and the role of the funders for the present study and, if applicable, for the original study on which the present article is based | Reported with submission |

*Give information separately for exposed and unexposed groups.

**Note:** An Explanation and Elaboration article discusses each checklist item and gives methodological background and published examples of transparent reporting. The STROBE checklist is best used in conjunction with this article (freely available on the Web sites of PLoS Medicine at http://www.plosmedicine.org/, Annals of Internal Medicine at http://www.annals.org/, and Epidemiology at http://www.epidem.com/). Information on the STROBE Initiative is available at http://www.strobe-statement.org.
