# Supplementary material for: Parental death in childhood and pathways to increased mortality across the life course in Stockholm, Sweden: A cohort study
Source: PLoS Med. 2021 Mar 11;18(3):e1003549. doi: 10.1371/journal.pmed.1003549 (PMC7951838; doi:10.1371/journal.pmed.1003549)
Supplement: S1 Protocol — (DOCX) [file pmed.1003549.s002.docx]

**S1 Protocol**

**An extract from the funding proposal to the Swedish Research Council (funded, Rostila, 2017-03266)**

**Aim and research questions**

The aim of the current project is to examine whether the death of a close family member in childhood influences mortality and disease in adulthood. We will in particular consider health consequences using a life course approach.

1. Is exposure to parental (…) death in childhood associated with mortality?
2. Do associations vary by gender, other socio-demographic factors and cause of death of both deceased individuals and their bereaved family members?
3. Can such associations be mediated by the individuals’ developmental life trajectories? (low school grades, criminality in adolescence and young adulthood, unemployment, low income etc.) (pathway)

**An extract from the protocol of analysis**

**Analytical plan**

1. Mortality of children who lost a parent in childhood/adolescence
   1. Exposure: parental death in period I-II
   2. Outcome: all-cause mortality (follow-up starts from either immediately after the period III or 1985, depending on variables involved)
   3. Covariates:
      1. Confounders: parental socioeconomic circumstances in 1953, child’s health status at birth (gestational age, birth weight) and/or before baseline
      2. Mediators: cognitive and psychological characteristics in conscription (around 1970), behaviours in 1985, socioeconomic circumstances in adulthood (income, education, social class)

**Changes from the plan**

- The information of income, education and social class in the early adulthood was available from data in 1980s and 1990, thus the follow-up was started from 1991.
- The contribution of gestational age, birth weight to the model was little while the use of these variables would have resulted in the loss of quite a few offspring due to missing data. These variables were decided not to be included.
- Familial and behavioural factors were obtained from Period III (ages 13-19) instead of data from the conscription examination or a survey on cultural and behavioural aspects in 1985. This is because the conscription data were available only in men and thus the use will result in lower power. Behavioural information measured in 1985 were considered to be less relevant to the association of our interest.
- During the process of revision of our manuscript, in response to a reviewer’s comment, generalized structural equation modelling, an analysis starting follow-up from 1973, and assessment of hazard ratios for concordant/discordant causes of death between the deceased parent and offspring have been added.
