# Supplementary material for: Parental death in childhood and pathways to increased mortality across the life course in Stockholm, Sweden: A cohort study
Source: PLoS Med. 2021 Mar 11;18(3):e1003549. doi: 10.1371/journal.pmed.1003549 (PMC7951838; doi:10.1371/journal.pmed.1003549)
Supplement: S1 Table — (DOCX) [file pmed.1003549.s003.docx]

**Content**

**T1.** Characteristics of offspring the death of a parent during childhood (age 0-12) and sex.

**T2.** Hazard ratios for the association between the death of a parent during childhood (age 0-12) and all-cause mortality from age 38 to 63.

**T3.** The test of interaction in the association between parental death and mortality by offspring’s sex.

**T4.** The difference between fathers’ and mothers’ death in the associations with mortality.

**T5.** Hazard ratios for the association between the death of a parent during childhood (age 0-12) and all-cause mortality from age 38 to 63, by the sex of deceased parent and offspring.

**T6.** Generalised structural equation modelling linking the death of a parent during childhood (age 0-12) and all-cause mortality (age 38-63).

**T7.** Multimediator analysis for Cox proportional hazard regression for the association between the death of a parent in childhood (age 0-12) and all-cause mortality (age 38-63), by sex of deceased parent and offspring.

**T8.** Hazard ratios for the association between the death of a parent during childhood (age 0-12) and all-cause mortality from age 20 to 63.

**T1.** Characteristics of offspring the death of a parent during childhood (age 0-12) and sex.

|  | **Male** | | | **Female** | | |
| --- | --- | --- | --- | --- | --- | --- |
|  | **No parental death** | **Parental death** | **p-value** | **No parental death** | **Parental death** | **p-value** |
|  | **N (%)*** | **N (%)*** |  | **N (%)*** | **N (%)*** |  |
| **Total** | **6,511 (97.1)** | **198 (3.0)** |  | **6,197 (97.1)** | **187 (2.9)** |  |
| **Confounding variables measured at birth** |  |  |  |  |  |  |
| **Birth order** |  |  |  |  |  |  |
| First-born | 3,762 (57.8) | 90 (45.5) |  | 3,539 (57.1) | 82 (43.9) |  |
| Later-born | 2,749 (42.2) | 108 (54.6) | 0.001 | 2,658 (42.9) | 105 (56.2) | <0.001 |
| **Parental social class** |  |  |  |  |  |  |
| Upper middle class and higher | 846 (13.0) | 31 (15.7) |  | 801 (12.9) | 30 (16.0) |  |
| Lower middle class, including officials and non-agricultural workers | 2,034 (31.2) | 59 (29.8) |  | 1,919 (31.0) | 59 (31.6) |  |
| Lower middle class, including self-employed | 393 (6.0) | 10 (5.1) |  | 348 (5.6) | 17 (9.1) |  |
| Working class, skilled workers | 1,784 (27.4) | 43 (21.7) |  | 1,795 (29.0) | 41 (21.9) |  |
| Working class, unskilled workers | 1,254 (19.3) | 48 (24.2) | 0.182 | 1,142 (18.4) | 36 (19.3) | 0.081 |
| Missing | 200 (3.1) | 7 (3.5) |  | 192 (3.1) | 4 (2.1) |  |
| **Mediators** |  |  |  |  |  |  |
| **Familial and behavioural factors (age 13-19)** |  |  |  |  |  |  |
| **Psychiatric problems in parents/surviving parent** |  |  |  |  |  |  |
| No | 6,334 (97.3) | 193 (97.5) |  | 6,024 (97.2) | 181 (96.8) |  |
| Yes | 177 (2.7) | 5 (2.5) | 0.869 | 173 (2.8) | 6 (3.2) | 0.734 |
| **Alcohol problems in parents/surviving parent** |  |  |  |  |  |  |
| No | 6,358 (97.7) | 197 (99.5) |  | 6,035 (97.4) | 185 (98.9) |  |
| Yes | 153 (2.4) | 1 (0.5) | 0.088 | 162 (2.6) | 2 (1.1) | 0.188 |
| **Receipt of social assistance** |  |  |  |  |  |  |
| No | 5,808 (89.2) | 171 (86.4) |  | 5,568 (89.9) | 151 (80.8) |  |
| Yes | 703 (10.8) | 27 (13.6) | 0.206 | 629 (10.2) | 36 (19.3) | <0.001 |
| **Delinquency**** |  |  |  |  |  |  |
| Mean (SD), 5th to 95th percentiles | 0.4 (0.9), 0 to 2 | 0.5 (0.9), 0 to 2 | 0.056 | 0.1 (0. 5), 0 to 1 | 0.1 (0.5), 0 to 1 | 0.1496 |
| **Socioeconomic factors (age 27-37)** |  |  |  |  |  |  |
| **Educational attainment** |  |  |  |  |  |  |
| Post-secondary | 2,285 (35.1) | 50 (25.3) |  | 2,383 (38.5) | 87 (46.5) |  |
| Upper secondary | 2,724 (41.8) | 100 (50.5) |  | 2,822 (45.5) | 67 (35.8) |  |
| Less than secondary | 1,452 (22.3) | 46 (23.2) | 0.012 | 960 (15.5) | 33 (17.7) | 0.025 |
| Missing | 50 (0.8) | 2 (1.0) |  | 32 (0.5) | 0 |  |
| **Social class** |  |  |  |  |  |  |
| Professional | 744 (11.4) | 19 (9.6) |  | 371 (6.0) | 9 (4.8) |  |
| Business owner | 325 (5.0) | 11 (5.6) |  | 95 (1.5) | 1 (0.5) |  |
| Mid-level office worker | 1,182 (18.2) | 24 (12.1) |  | 1,195 (19.3) | 43 (23.0) |  |
| Lower-level office worker | 797 (12.2) | 14 (7.1) |  | 1,529 (24.7) | 34 (18.2) |  |
| Skilled production/service worker | 1,166 (17.9) | 36 (18.2) |  | 455 (7.3) | 14 (7.5) |  |
| Unskilled production/service worker | 1,290 (19.8) | 51 (25.8) |  | 933 (15.1) | 36 (19.3) |  |
| Other *** | 997 (15.3) | 43 (21.7) | 0.008 | 1,603 (25.9) | 50 (26.7) | 0.225 |
| Missing | 10 (0.2) | 0 |  | 16 (0.3) | 0 |  |
| **Income (1000 Swedish crown)** |  |  |  |  |  |  |
| Mean (SD), 5 to 95 percentiles (per 1,000 Swedish krona) | 114.8 (56.9),10.8 to 206.4 | 103.2 (64.3), 0 to 188.4 | 0.004 | 74.7 (39.1) | 74.5 (38.8) | 0.990 |
| Missing | 16 (0.3) | 0 |  | 22 (0.4) | 0 |  |

*Delinquency and income in adulthood are continuous variables and mean (SD) and 5th and 95th percentiles are displayed.

** Delinquency was defined by the number of welfare support decisions by the Child Welfare Committee from age 13 to 19. The values ranged from 0 to 9.

*** Other included unclassified employees, pensioners, homemakers (male and female), students, part-time workers (less than 16h/week), and other unspecified groups.

P-value refers to Chi-square test for categorical variables and Wilcoxon rank-sum test for continuous variables. All tests were conducted after excluding missing data.

**T2.** Hazard ratios for the association between the death of a parent during childhood (age 0-12) and all-cause mortality from age 38 to 63.

|  | **Event* / N** | **Model 1** | **Model 2** | **Model 3** | **Model 4** | **Model 5** | **Model 6** | **Model 7** | **Model 8** | **Model 9** | **Model 10** | **Model 11** |
| --- | --- | --- | --- | --- | --- | --- | --- | --- | --- | --- | --- | --- |
|  |  | **HR (95% CI), p-value** | **HR (95% CI), p-value** | **HR (95% CI), p-value** | **HR (95% CI), p-value** | **HR (95% CI), p-value** | **HR (95% CI), p-value** | **HR (95% CI), p-value** | **HR (95% CI), p-value** | **HR (95% CI), p-value** | **HR (95% CI), p-value** | **HR (95% CI), p-value** |
| **All offspring** |  |  |  |  |  |  |  |  |  |  |  |  |
| **Death of a parent** | 935 / 12,582 |  |  |  |  |  |  |  |  |  |  |  |
| No parental death | 895 / 12,210 | Reference | Reference | Reference | Reference | Reference | Reference | Reference | Reference | Reference | Reference | Reference |
| Parental death | 40 / 372 | 1.50 (1.09, 2.05), 0.013 | 1.52 (1.10, 2.08), 0.010 | 1.53 (1.11, 2.10), 0.009 | 1.49 (1.08, 2.04), 0.015 | 1.39 (1.01, 1.91), 0.044 | 1.49 (1.09, 2.05), 0.013 | 1.38 (1.01, 1.90), 0.046 | 1.39 (1.01, 1.91), 0.045 | 1.34 (0.98, 1.85), 0.070 | 1.31 (0.96, 1.81), 0.092 | 1.32 (0.96, 1.81), 0.091 |

*Event: Death of offspring.

In the analyses of the death of a father, those who experienced a death of a mother were excluded. In the analyses of the death of a mother, those who experienced the death of a father were excluded.

Model 1: Unadjusted

Model 2: Adjusted for covariates (sex of offspring, birth order, parental social class) (in models stratified by the sex of offspring, sex was not adjusted for)

Model 3: Model 2 + psychiatric problems and alcohol problems in the parents/remaining parent (age 13-19)

Model 4: Model 2 + the receipt of social assistance (age 13-19)

Model 5: Model 2 + delinquency (age 13-19)

Model 6: Model 2 + psychiatric problems and alcohol problems in the parents/remaining parent + the receipt of social assistance (age 13-19)

Model 7: Model 2 + psychiatric problems and alcohol problems in the parents/remaining parent + the receipt of social assistance + delinquency (age 13-19)

Model 8: Model 7 + education (age 37)

Model 9: Model 7 + social class (age 27)

Model 10: Model 7 + income (age 32)

Model 11: Model 7 + education (age 37) + social class (age 27) + income (age 32)

**T3.** The test of interaction in the association between parental death and mortality by offspring’s sex.

|  | **Unadjusted HR (95% CI), p-value** |
| --- | --- |
| **Parental death** |  |
| No parental death | Reference |
| Parental death | 1.57 (1.07, 2.32), 0.021 |
| **Offspring’s sex** |  |
| Male | Reference |
| Female | 0.64 (0.56, 0.73), <0.001 |
| **Parental death * offspring’s sex** | 0.76 (0.39, 1.50), 0.435 |

**T4.** The difference between fathers’ and mothers’ death in the associations with mortality.

|  | **Unadjusted HR (95% CI), p-value** |
| --- | --- |
| **Parental death** |  |
| No parental death | 0.67 (0.47, 0.97), 0.036 |
| Father’s death | Reference |
| Mother’s death | 0.87 (0.44, 1.75), 0.705 |

**T5.** Hazard ratios for the association between the death of a parent during childhood (age 0-12) and all-cause mortality from age 38 to 63, by the sex of deceased parent and offspring.

|  | **Event* / N** | **Model 1** | **Model 2** | **Model 3** | **Model 4** | **Model 5** | **Model 6** | **Model 7** | **Model 8** | **Model 9** | **Model 10** | **Model 11** |
| --- | --- | --- | --- | --- | --- | --- | --- | --- | --- | --- | --- | --- |
|  |  | **HR (95% CI), p-value** | **HR (95% CI), p-value** | **HR (95% CI), p-value** | **HR (95% CI), p-value** | **HR (95% CI), p-value** | **HR (95% CI), p-value** | **HR (95% CI), p-value** | **HR (95% CI), p-value** | **HR (95% CI), p-value** | **HR (95% CI), p-value** | **HR (95% CI), p-value** |
| **Stratified by sex of offspring** | | | | | | | | | | | | |
| **Male offspring** | 575 / 6,439 |  |  |  |  |  |  |  |  |  |  |  |
| No parental death | 548 / 6,250 | Reference | Reference | Reference | Reference | Reference | Reference | Reference | Reference | Reference | Reference | Reference |
| Parental death | 27 / 189 | 1.68 (1.14, 2.47), 0.009 | 1.69 (1.15, 2.49), 0.008 | 1.71 (1.16, 2.52), 0.007 | 1.68 (1.14, 2.47), 0.009 | 1.53 (1.04, 2.26), 0.032 | 1.70 (1.15, 2.50), 0.008 | 1.54 (1.04, 2.27), 0.030 | 1.50 (1.02, 2.21), 0.042 | 1.45 (0.99, 2.15), 0.060 | 1.39 (0.94, 2.06), 0.097 | 1.37 (0.93, 2.03), 0.111 |
| **Female offspring** | 360 / 6,143 |  |  |  |  |  |  |  |  |  |  |  |
| No parental death | 347 / 5,960 | Reference | Reference | Reference | Reference | Reference | Reference | Reference | Reference | Reference | Reference | Reference |
| Parental death | 13 / 183 | 1.23 (0.71, 2.14), 0.460 | 1.27 (0.73, 2.21), 0.399 | 1.27 (0.73, 2.21), 0.404 | 1.21 (0.69, 2.11), 0.506 | 1.22 (0.70, 2.13), 0.481 | 1.20 (0.69, 2.10), 0.517 | 1.18 (0.67, 2.06), 0.561 | 1.18 (0.67, 2.07), 0.560 | 1.21 (0.69, 2.11), 0.513 | 1.20 (0.68, 2.09), 0.530 | 1.19 (0.68, 2.08), 0.552 |
| **By sex of the deceased parent** | | | | | | | | | | | | |
| **Death of the father** | 924 / 12,469 |  |  |  |  |  |  |  |  |  |  |  |
| No parental death | 895 / 12,210 | Reference | Reference | Reference | Reference | Reference | Reference | Reference | Reference | Reference | Reference | Reference |
| Parental death | 29 / 259 | 1.57 (1.08, 2.27), 0.017 | 1.58 (1.09, 2.29), 0.015 | 1.58 (1.09, 2.29), 0.016 | 1.54 (1.06, 2.23), 0.023 | 1.47 (1.02, 2.14), 0.040 | 1.55 (1.07, 2.24), 0.021 | 1.47 (1.02, 2.14), 0.041 | 1.47 (1.01, 2.13), 0.043 | 1.45 (1.00, 2.10), 0.051 | 1.42 (0.98, 2.06), 0.062 | 1.42 (0.98, 2.06), 0.063 |
| **Death of the mother** | 906 / 12,323 |  |  |  |  |  |  |  |  |  |  |  |
| No parental death | 895 / 12,210 | Reference | Reference | Reference | Reference | Reference | Reference | Reference | Reference | Reference | Reference | Reference |
| Parental death | 11 / 113 | 1.34 (0.74, 2.42), 0.339 | 1.37 (0.75, 2.48), 0.303 | 1.39 (0.76, 2.52), 0.281 | 1.36 (0.75, 2.47), 0.307 | 1.20 (0.66, 2.17), 0.556 | 1.38 (0.76, 2.51), 0.287 | 1.21 (0.67, 2.19), 0.536 | 1.23 (0.68, 2.24), 0.492 | 1.15 (0.63, 2.09), 0.644 | 1.11 (0.61, 2.01), 0.735 | 1.12 (0.62, 2.04), 0.703 |
| **By sex of the deceased parent and offspring** | | | | | | | | | | | | |
| **Death of the father** |  |  |  |  |  |  |  |  |  |  |  |  |
| **Male offspring** | 565 / 6,384 |  |  |  |  |  |  |  |  |  |  |  |
| No parental death | 548 / 6,250 | Reference | Reference | Reference | Reference | Reference | Reference | Reference | Reference | Reference | Reference | Reference |
| Parental death | 17 / 134 | 1.49 (0.92, 2.42), 0.105 | 1.52 (0.94, 2.47), 0.089 | 1.52 (0.94, 2.46), 0.090 | 1.51 (0.93, 2.44), 0.097 | 1.43 (0.88, 2.32), 0.149 | 1.51 (0.93, 2.45), 0.095 | 1.43 (0.88, 2.32), 0.147 | 1.38 (0.85, 2.24), 0.195 | 1.38 (0.85, 2.24), 0.196 | 1.34 (0.82, 2.17), 0.241 | 1.31 (0.80, 2.12), 0.281 |
| **Female offspring** | 359 / 6,085 |  |  |  |  |  |  |  |  |  |  |  |
| No parental death | 347 / 5,960 | Reference | Reference | Reference | Reference | Reference | Reference | Reference | Reference | Reference | Reference | Reference |
| Parental death | 12 / 125 | 1.68 (0.95, 2.99), 0.076 | 1.73 (0.97, 3.08), 0.062 | 1.71 (0.96, 3.05), 0.067 | 1.62 (0.91, 2.88), 0.104 | 1.64 (0.92, 2.93), 0.095 | 1.63 (0.91, 2.91), 0.098 | 1.60 (0.90, 2.87), 0.112 | 1.60 (0.89, 2.86), 0.117 | 1.64 (0.92, 2.93), 0.095 | 1.62 (0.91, 2.90), 0.103 | 1.60 (0.89, 2.87), 0.114 |
| **Death of the mother** | 558 / 6,305 |  |  |  |  |  |  |  |  |  |  |  |
| **Male offspring** |  |  |  |  |  |  |  |  |  |  |  |  |
| No parental death | 548 / 6,250 | Reference | Reference | Reference | Reference | Reference | Reference | Reference | Reference | Reference | Reference | Reference |
| Parental death | 10 / 55 | 2.13 (1.14, 3.99), 0.018 | 2.10 (1.12, 3.93), 0.020 | 2.11 (1.13, 3.95), 0.020 | 2.10 (1.12, 3.93), 0.020 | 1.74 (0.93, 3.26), 0.084 | 2.10 (1.12, 3.93), 0.020 | 1.74 (0.93, 3.26), 0.084 | 1.76 (0.94, 3.29), 0.079 | 1.60 (0.85, 3.00), 0.143 | 1.49 (0.79, 2.80), 0.216 | 1.51 (0.80, 2.83), 0.202 |
| **Female offspring** | 348 / 6,018 |  |  |  |  |  |  |  |  |  |  |  |
| No parental death | 347 / 5,960 | Reference | Reference | Reference | Reference | Reference | Reference | Reference | Reference | Reference | Reference | Reference |
| Parental death | 1 / 58 | 0.29 (0.04, 2.08), 0.220 | 0.30 (0.04, 2.14), 0.231 | 0.31 (0.04, 2.18), 0.238 | 0.30 (0.04, 2.13), 0.228 | 0.30 (0.04, 2.13), 0.229 | 0.31 (0.04, 2.21), 0.243 | 0.31 (0.04, 2.20), 0.241 | 0.32 (0.04, 2.25), 0.250 | 0.31 (0.04, 2.24), 0.249 | 0.31 (0.04, 2.24), 0.248 | 0.32 (0.04, 2.26), 0.251 |

*Event: Death of offspring.

In the analyses of the death of a father, those who experienced a death of a mother were excluded. In the analyses of the death of a mother, those who experienced the death of a father were excluded.

Model 1: Unadjusted

Model 2: Adjusted for covariates (sex of offspring, birth order, parental social class) (in models stratified by the sex of offspring, sex was not adjusted for)

Model 3: Model 2 + psychiatric problems and alcohol problems in the parents/remaining parent (age 13-19)

Model 4: Model 2 + the receipt of social assistance (age 13-19)

Model 5: Model 2 + delinquency (age 13-19)

Model 6: Model 2 + psychiatric problems and alcohol problems in the parents/remaining parent + the receipt of social assistance (age 13-19)

Model 7: Model 2 + psychiatric problems and alcohol problems in the parents/remaining parent + the receipt of social assistance + delinquency (age 13-19)

Model 8: Model 7 + education (age 37)

Model 9: Model 7 + social class (age 27)

Model 10: Model 7 + income (age 32)

Model 11: Model 7 + education (age 37) + social class (age 27) + income (age 32)

**T6.** Multimediator analysis for Cox proportional hazard regression for the association between the death of a parent in childhood (age 0-12) and all-cause mortality (age 38-63), by sex of deceased parent and offspring.

|  | **All offspring** | | **Male offspring** | **Female offspring** |
| --- | --- | --- | --- | --- |
|  | **Death of the father** | **Death of the mother** | **Death of a parent** | **Death of a parent** |
|  | **HR (95% CI), p-value** | **HR (95% CI), p-value** | **HR (95% CI), p-value** | **HR (95% CI), p-value** |
| **Total effect of the exposure** | 1.51 (1.04, 2.19), p=0.031 | 1.28 (0.71, 2.34), p=0.413 | 1.58 (1.07, 2.35), p=0.022 | 1.27 (0.73, 2.21), p=0.401 |
| **Path 1**: The effect of E that involve M1 (delinquency) and possibly M2 (adulthood income) | 1.02 (0.99, 1.06), p=0.151 | 1.05 (1.00, 1.10), p=0.059 | 1.04 (1.00, 1.09), p=0.071 | 1.02 (0.99, 1.05), p=0.222 |
| **Path 2**: The effect of E on Y only mediated through adulthood income | 1.03 (0.99, 1.06), p=0.141 | 1.06 (1.00, 1.12), p=0.056 | 1.08 (1.03, 1.14), p=0.006 | 1.00 (0.97, 1.03), p=0.974 |
| **Path 3**: The effect of E on Y not via pathways involving delinquency or income | 1.44 (0.99, 2.08), p=0.055 | 1.16 (0.64, 2.10), p=0.628 | 1.40 (0.95, 2.07), p=0.087 | 1.25 (0.72, 2.17), p=0.437 |

In the mediation analysis, it was not possible to model the non-linear relationship between delinquency and mortality, thus the association was treated as linear.

**T7.** Generalised structural equation modelling linking the death of a parent during childhood (age 0-12) and all-cause mortality (age 38-63).

| **PARENTAL DEATH (logistic regression)** | **Odds ratio (95% CI), p-value** |
| --- | --- |
| **Parental social class** |  |
| Upper middle class and higher | Reference |
| Lower middle class, including officials and non-agricultural workers | 0.81 (0.59, 1.10), 0.178 |
| Lower middle class, including self-employed | 0.99 (0.62, 1.57), 0.955 |
| Working class, skilled workers | 0.62 (0.44, 0.86), 0.005 |
| Working class, unskilled workers | 0.95 (0.68, 1.32), 0.745 |
| **DELINQUENCY (linear regression)** | **Coefficient (95% CI), p-value** |
| **Parental death** |  |
| No parental death | Reference |
| Parental death | 0.08 (0.01, 0.15), 0.025 |
| **Sex of offspring** |  |
| Male | Reference |
| Female | -0.24 (-0.27, -0.22), <0.001 |
| **Birth order** |  |
| First-born | Reference |
| Later-born | 0.06 (0.04, 0.09), <0.001 |
| **Parental social class** |  |
| Upper middle class and higher | Reference |
| Lower middle class, including officials and non-agricultural workers | 0.10 (0.06, 0.14), <0.001 |
| Lower middle class, including self-employed | 0.12 (0.06, 0.18), <0.001 |
| Working class, skilled workers | 0.22 (0.18, 0.26), <0.001 |
| Working class, unskilled workers | 0.27 (0.22, 0.31), <0.001 |
| **EDUCATIONAL ATTAINMENT (multinomial logistic regression)** | **Risk ratio (95% CI), p-value** |
| **1. Post-secondary (reference)** |  |
| **2. Upper secondary** |  |
| **Parental death** |  |
| No parental death | Reference |
| Parental death | 1.06 (0.83, 1.35), 0.652 |
| **Sex of offspring** |  |
| Male | Reference |
| Female | 1.02 (0.94, 1.11), 0.576 |
| **Birth order** |  |
| First-born | Reference |
| Later-born | 1.21 (1.11, 1.31), <0.001 |
| **Parental social class** |  |
| Upper middle class and higher | Reference |
| Lower middle class, including officials and non-agricultural workers | 2.33 (2.04, 2.65), <0.001 |
| Lower middle class, including self-employed | 2.61 (2.15, 3.17), <0.001 |
| Working class, skilled workers | 4.54 (3.96, 5.20), <0.001 |
| Working class, unskilled workers | 6.64 (5.69, 7.75), <0.001 |
| **Delinquency** | 2.20 (1.97, 2.47), <0.001 |
| **3. Less than secondary** |  |
| **Parental death** |  |
| No parental death | Reference |
| Parental death | 1.11 (0.81, 1.51), 0.523 |
| **Sex of offspring** |  |
| Male | Reference |
| Female | 0.73 (0.66, 0.82), <0.001 |
| **Birth order** |  |
| First-born | Reference |
| Later-born | 1.32 (1.19, 1.47), <0.001 |
| **Parental social class** |  |
| Upper middle class and higher | Reference |
| Lower middle class, including officials and non-agricultural workers | 3.03 (2.44, 3.76), <0.001 |
| Lower middle class, including self-employed | 4.26 (3.21, 5.66), <0.001 |
| Working class, skilled workers | 7.81 (6.29, 9.70), <0.001 |
| Working class, unskilled workers | 13.89 (11.05, 17.45), <0.001 |
| **Delinquency** | 2.85 (2.53, 3.20), <0.001 |
| **SOCIAL CLASS (multinomial logistic regression)** | **Risk ratio (95% CI), p-value** |
| **1. High (reference)** |  |
| **2. Office worker** |  |
| **Parental death** |  |
| No parental death | Reference |
| Parental death | 0.89 (0.61, 1.31), 0.562 |
| **Sex of offspring** |  |
| Male | Reference |
| Female | 3.27 (2.88, 3.72), <0.001 |
| **Birth order** |  |
| First-born | Reference |
| Later-born | 1.10 (0.97, 1.24), 0.132 |
| **Parental social class** |  |
| Upper middle class and higher | Reference |
| Lower middle class, including officials and non-agricultural workers | 1.77 (1.51, 2.09), <0.001 |
| Lower middle class, including self-employed | 1.51 (1.15, 1.96), 0.003 |
| Working class, skilled workers | 2.14 (1.78, 2.58), <0.001 |
| Working class, unskilled workers | 2.67 (2.12, 3.35), <0.001 |
| **Delinquency** | 0.98 (0.85, 1.13), 0.737 |
| **Educational attainment** |  |
| Post-secondary | Reference |
| Upper secondary | 2.52 (2.18, 2.91), <0.001 |
| Less than secondary | 1.48 (1.21, 1.80), <0.001 |
| **3. Manual worker and other** |  |
| **Parental death** |  |
| No parental death | Reference |
| Parental death | 1.27 (0.88, 1.82), 0.205 |
| **Sex of offspring** |  |
| Male | Reference |
| Female | 2.40 (2.12, 2.73), <0.001 |
| **Birth order** |  |
| First-born | Reference |
| Later-born | 1.13 (1.00, 1.28), 0.046 |
| **Parental social class** |  |
| Upper middle class and higher | Reference |
| Lower middle class, including officials and non-agricultural workers | 1.47 (1.24, 1.73), <0.001 |
| Lower middle class, including self-employed | 1.55 (1.19, 2.01), 0.001 |
| Working class, skilled workers | 2.31 (1.92, 2.78), <0.001 |
| Working class, unskilled workers | 3.29 (2.62, 4.12), <0.001 |
| **Delinquency** | 1.48 (1.30, 1.68), <0.001 |
| **Educational attainment** |  |
| Post-secondary | Reference |
| Upper secondary | 5.19 (4.49, 5.99), <0.001 |
| Less than secondary | 4.93 (4.08, 5.96), <0.001 |
| **INCOME (linear regression)** | **Coefficient (95% CI), p-value** |
| **Parental death** |  |
| No parental death | Reference |
| Parental death | -2.58 (-7.30, 2.13), 0.282 |
| **Sex of offspring** |  |
| Male | Reference |
| Female | -42.63 (-44.28, -40.99), <0.001 |
| **Birth order** |  |
| First-born | Reference |
| Later-born | -1.91 (-3.53, -0.29), 0.021 |
| **Parental social class** |  |
| Upper middle class and higher | Reference |
| Lower middle class, including officials and non-agricultural workers | -0.98 (-3.61, 1.65), 0.464 |
| Lower middle class, including self-employed | 0.17 (-3.77, 4.11), 0.932 |
| Working class, skilled workers | -0.44 (-3.18, 2.30), 0.753 |
| Working class, unskilled workers | -0.22 (-3.20, 2.75), 0.883 |
| **Delinquency** | -7.26 (-8.43, -6.09), <0.001 |
| **Educational attainment** |  |
| Post-secondary | Reference |
| Upper secondary | -9.10 (-11.01, -7.19), <0.001 |
| Less than secondary | -14.51 (-16.96, -12.06), <0.001 |
| **Social class** |  |
| High | Reference |
| Office worker | -10.71 (-13.43, -7.99), <0.001 |
| Manual worker and other | -34.20 (-36.90, -31.50), <0.001 |
| **MORTALITY (parametric survival regression)** | **Hazard ratio (95% CI), p-value** |
| **Parental death** |  |
| No parental death | Reference |
| Parental death | 1.02 (0.92, 1.13), 0.739 |
| **Sex of offspring** |  |
| Male | Reference |
| Female | 0.96 (0.92, 1.00), 0.059 |
| **Birth order** |  |
| First-born | Reference |
| Later-born | 0.99 (0.96, 1.03), 0.616 |
| **Parental social class** |  |
| Upper middle class and higher | Reference |
| Lower middle class, including officials and non-agricultural workers | 1.00 (0.95, 1.06), 0.961 |
| Lower middle class, including self-employed | 0.99 (0.91, 1.08), 0.853 |
| Working class, skilled workers | 0.97 (0.91, 1.03), 0.318 |
| Working class, unskilled workers | 0.97 (0.91, 1.03), 0.343 |
| **Delinquency** | 1.06 (1.03, 1.09), <0.001 |
| **Educational attainment** |  |
| Post-secondary | Reference |
| Upper secondary | 1.01 (0.97, 1.05), 0.755 |
| Less than secondary | 1.05 (0.99, 1.10), 0.108 |
| **Social class** |  |
| High | Reference |
| Office worker | 0.99 (0.93, 1.05), 0.802 |
| Manual worker and other | 1.00 (0.94, 1.07), 0.927 |
| **Income** | 0.9996 (0.9992, 0.99999), 0.046 |

Variable displayed by upper cases indicate the outcome. Social class was grouped into three levels: high (professional and business owner), office worker (mid- and lower-level office worker) and manual and other (skilled and unskilled production/service worker and other). CI: confidence interval.

**T8.** Hazard ratios for the association between the death of a parent during childhood (age 0-12) and all-cause mortality from age 20 to 63.

|  | **Event* / N** | **Model 1** | **Model 2** | **Model 3** | **Model 4** | **Model 5** | **Model 6** | **Model 7** |
| --- | --- | --- | --- | --- | --- | --- | --- | --- |
|  |  | **HR (95% CI)**  **p-value** | **HR (95% CI)**  **p-value** | **HR (95% CI)**  **p-value** | **HR (95% CI)**  **p-value** | **HR (95% CI)**  **p-value** | **HR (95% CI)**  **p-value** | **HR (95% CI)**  **p-value** |
| **The death of a parent** | 1,141 / 13,407 |  |  |  |  |  |  |  |
| No parental death | 1,096 / 13,014 | Reference | Reference | Reference | Reference | Reference | Reference | Reference |
| Parental death | 45 / 393 | 1.37 (1.02, 1.84)  0.039 | 1.38 (1.02, 1.86), 0.035 | 1.39 (1.03, 1.88), 0.030 | 1.34 (0.99, 1.81), 0.055 | 1.27 (0.94, 1.71), 0.120 | 1.36 (1.01, 1.83), 0.046 | 1.27 (0.94, 1.71), 0.120 |

*Event: Death of offspring.

In the analyses of death of the father, those who experienced death of the mother were excluded. In the analyses of death of the mother, those who experienced death of the father were excluded.

Model 1: Unadjusted

Model 2: Adjusted for covariates (sex of offspring, birth order, social class)

Model 3: Model 2 + psychiatric problems and alcohol problems in the parents/remaining parent (age 13-19)

Model 4: Model 2 + the receipt of social assistance (age 13-19)

Model 5: Model 2 + delinquency (age 13-19)

Model 6: Model 2 + psychiatric problems and alcohol problems in the parents/remaining parent + the receipt of social assistance (age 13-19)

Model 7: Model 2 + psychiatric problems and alcohol problems in the parents/remaining parent + the receipt of social assistance + delinquency (age 13-19)
