# Supplementary material for: Parental death in childhood and pathways to increased mortality across the life course in Stockholm, Sweden: A cohort study
Source: PLoS Med. 2021 Mar 11;18(3):e1003549. doi: 10.1371/journal.pmed.1003549 (PMC7951838; doi:10.1371/journal.pmed.1003549)
Supplement: S1 Fig — (DOCX) [file pmed.1003549.s004.docx]

**Coefficients, risk ratios and hazard ratios estimated using GSEM linking parental death (age 0-12­) to mortality (age 38-63).**

 Distribution and link function used for regression models are displayed in each box. Statistically significant coefficients relevant to direct and indirect paths are shown, and all coefficients are reported in a supplementary table (T7 in S1 Table). Social class was grouped into three levels: high (professional and business owner), office worker (mid- and lower-level office worker) and manual and other (skilled and unskilled production/service worker and other). Highest education and social class were used as references in the multinomial logistic regressions. β: coefficient, RR: relative risk, HR: hazard ratio.

**Interpretation**

Parental death was associated with a 0.08 higher delinquency score, and a one score increase in delinquency was associated with a higher likelihood of upper secondary or less than secondary education compared to post-secondary education (RRs 2.20 and 2.85, respectively). Delinquency and lower education level were associated with higher likelihood of office worker (including mid-and lower level office workers) and manual and other workers (including skilled and unskilled production/service workers and other), compared to the likelihood of high social class (including professional and business owner), and these characteristics in turn were associated with lower income. Finally, a one-unit increase in delinquency and income showed statistically significant associations with mortality (HRs 1.06 and 0.9996, respectively).
